# Supplementary material for: Comment on "Magnetosensitive neurons mediate geomagnetic orientation in Caenorhabditis elegans"
Source: eLife. 2018 Apr 13;7:e30187. doi: 10.7554/eLife.30187 (PMC5898909; doi:10.7554/eLife.30187)
Supplement: Transparent reporting form [file elife-30187-transrepform.docx]

***eLife’s* transparent reporting form**

We encourage authors to provide detailed information *within their submission* to facilitate the interpretation and replication of experiments. Authors can upload supporting documentation to indicate the use of appropriate reporting guidelines for health-related research (see [EQUATOR Network](http://www.equator-network.org/%20)), life science research (see the [BioSharing Information Resource](https://biosharing.org/)), or the [ARRIVE guidelines](http://www.plosbiology.org/article/info:doi/10.1371/journal.pbio.1000412) for reporting work involving animal research. Where applicable, authors should refer to any relevant reporting standards documents in this form.

If you have any questions, please consult our Journal Policies and/or contact us: [editorial@elifesciences.org](mailto:editorial@elifesciences.org).

**Sample-size estimation**

- You should state whether an appropriate sample size was computed when the study was being designed
- You should state the statistical method of sample size computation and any required assumptions
- If no explicit power analysis was used, you should describe how you decided what sample (replicate) size (number) to use

Please outline where this information can be found within the submission (e.g., sections or figure legends), or explain why this information doesn’t apply to your submission:

In performing our experiments we chose n numbers that were analogous to those performed by Vidal-Gadea. For instance, when performing the burrowing assay with vertically aligned pipettes Vidal-Gadea used an n between 9 and 30. We used a n between 20 and 40.

**Replicates**

- You should report how often each experiment was performed
- You should include a definition of biological versus technical replication
- The data obtained should be provided and sufficient information should be provided to indicate the number of independent biological and/or technical replicates
- If you encountered any outliers, you should describe how these were handled
- Criteria for exclusion/inclusion of data should be clearly stated
- High-throughput sequence data should be uploaded before submission, with a private link for reviewers provided (these are available from both GEO and ArrayExpress)

Please outline where this information can be found within the submission (e.g., sections or figure legends), or explain why this information doesn’t apply to your submission:

We define a true replicate as a treatment application on one (independent) experimental unit (e.g. plate of worms), in contrast to a technical replicate which simply involves multiple measurements of the same experimental unit. This is stated explicitly in the manuscript. All n numbers in the manuscript represent biological replicates.

The magnetotaxis assay was performed on two separate occasions, one prior to visiting the Vidal-Gadea Lab and a larger test series afterwards. For the latter experiment we adapted our protocols based on Vidal-Gadeas suggestions (e.g. 30mins testing time v. 60mins testing time, placement of magnets). In this manuscript we present the data from the second experiment. In neither of these two experiments did we observe a significant effect of the magnetic stimulus. The magnetite-contamination magnet assay was performed one time. The burrowing assay was performed twice, once using starved animals and once with starved and fed animals. For the purposes of this manuscript we only present the latter in the paper. In neither case did we observe a significant magnetic effect. The horizontal plate assay was performed twice. Blind analysis of our first experiment revealed that worms had a topographic preference, which we attributed to a very subtle odor/air flow in the magnetically shielded room. This data was therefore excluded, the room was re-built, the air flow modified, and the experiment repeated. In this second experiment we observed no topographic preferences, and we therefore present this experiment.

**Statistical reporting**

- Statistical analysis methods should be described and justified
- Raw data should be presented in figures whenever informative to do so (typically when N per group is less than 10)
- For each experiment, you should identify the statistical tests used, exact values of N, definitions of center, methods of multiple test correction, and dispersion and precision measures (e.g., mean, median, SD, SEM, confidence intervals; and, for the major substantive results, a measure of effect size (e.g., Pearson's r, Cohen's d)
- Report exact p-values wherever possible alongside the summary statistics and 95% confidence intervals. These should be reported for all key questions and not only when the p-value is less than 0.05.

Please outline where this information can be found within the submission (e.g., sections or figure legends), or explain why this information doesn’t apply to your submission:

Statistical methods are described in the manuscript. To show individual biological replicates we have employed dot plots for all our graphs, with error bars. In each case we have indicated the statistical test employed, the sample size, and p-values. For ease of reference this is summarized in Supplementary Table 1.

(For large datasets, or papers with a very large number of statistical tests, you may upload a single table file with tests, Ns, etc., with reference to sections in the manuscript.)

**Group allocation**

- Indicate how samples were allocated into experimental groups (in the case of clinical studies, please specify allocation to treatment method); if randomization was used, please also state if restricted randomization was applied
- Indicate if masking was used during group allocation, data collection and/or data analysis

Please outline where this information can be found within the submission (e.g., sections or figure legends), or explain why this information doesn’t apply to your submission:

N/A

**Additional data files (“source data”)**

- We encourage you to upload relevant additional data files, such as numerical data that are represented as a graph in a figure, or as a summary table
- Where provided, these should be in the most useful format, and they can be uploaded as “Source data” files linked to a main figure or table
- Include model definition files including the full list of parameters used
- Include code used for data analysis (e.g., R, MatLab)
- Avoid stating that data files are “available upon request”

Please indicate the figures or tables for which source data files have been provided:

We included a supplementary table that summarises our statistical analysis.
